# Successful Treatment of Rapid Cycling Bipolar Disorder With Lurasidone, Clozapine, and Valproate: A Case Report

**DOI:** 10.1111/bdi.70155

**Published:** 2026-07-16

**Authors:** Petr Potměšil, Miloslav Kopeček, Veronika Andrashko

**Affiliations:** ^1^ Department of Pharmacology, Third Faculty of Medicine Charles University Prague 10 Czech Republic; ^2^ National Institute of Mental Health Klecany Czech Republic; ^3^ Department of Psychiatry, Third Faculty of Medicine Charles University Prague 10 Czech Republic

## 1 Case Presentation

We report on the case of a 30‐year‐old woman suffering from rapid cycling bipolar affective disorder type 1 (BD‐1) without psychotic features. Her first manic episode occurred when she was 14 years old. She was successfully treated with olanzapine and valproate. Since being diagnosed, she has been hospitalized five times for her BD‐1; for the past three years, she has been successfully stabilized on valproate, quetiapine, and aripiprazole. Her recent episode of rapid cycling has persisted for nine months, commencing after the discontinuation of valproate in preparation for a planned pregnancy. She has experienced recurrent episodes of depression and mania/hypomania, each lasting 1–2 weeks. During one depressive phase, she attempted suicide by strangulation. Treatment with lamotrigine (200 mg), lithium (1500 mg (plasma concentration 0.96 mmol/L)), quetiapine (600 mg), and long‐acting (monthly) intramuscular injections of aripiprazole (400 mg) was unsuccessful, as well as treatment with olanzapine, carbamazepine, and electroconvulsive therapy; these failures led to her being hospitalized for mania.

Post‐admission laboratory findings found normal liver, kidney, and thyroid function; toxicology screening tests were negative. Blood pressure, glycemic, and lipid parameters were normal. Magnetic resonance imaging excluded structural changes in the CNS.

**FIGURE 1:**
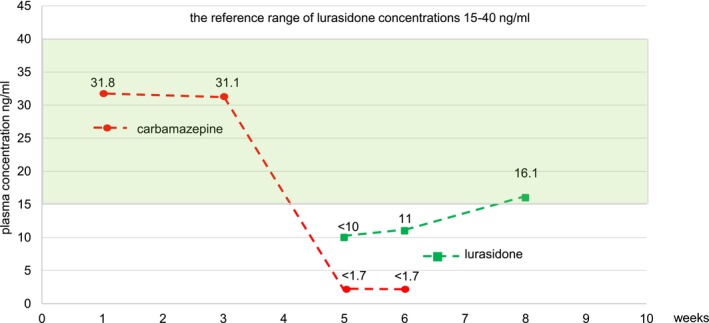
Carbamazepine–lurasidone interaction.

Before hospital admission, the patient was treated daily (q.d.) with vortioxetine (10 mg), olanzapine (10 mg), and carbamazepine (600 mg). Due to severe insomnia and hyperactivity, her pharmacotherapy was changed during her first hospital week. Vortioxetine was withdrawn, clonazepam was added for two weeks, and the patient began treatment with lurasidone (taken with meals), which was titrated from 37.5 mg q.d. up to 148 mg q.d. by the end of week three. Therapeutic lurasidone concentrations were not achieved due to increased lurasidone metabolism caused by a carbamazepine‐induced increase in the cytochrome 450 3A4 enzyme. Carbamazepine was, therefore, withdrawn at the end of week four and immediately switched to valproate. During week five, plasma lurasidone levels were < 10 ng/mL but increased to 11 ng/mL during week six. Lurasidone was maintained at 74 mg q.d. and olanzapine was replaced by clozapine during week five. Clozapine was titrated to 150 mg q.d. during week five and increased to 200 mg q.d. in week six; the plasma clozapine concentration was 251 ng/mL. Hematological monitoring confirmed clozapine therapy safety. During week eight, lurasidone concentrations (third measurement) were found to have increased to 16.1 ng/mL. After eleven weeks of hospitalization, the patient was released to outpatient care; she was, of course, informed about the pregnancy risk associated with valproate. Outpatient treatment included valproate (500 mg twice daily), lurasidone (74 mg q.d.), and clozapine (150 mg q.d.). This combination led to improvement in and stabilization of the patient's mood. Follow‐up showed no hospitalizations in the two years since discharge; she continues with clozapine and valproate treatment. The course of pharmacotherapy and results of therapeutic drug monitoring are presented in Table 1 and Figure 1.

**TABLE 1:** Patient pharmacotherapy and results of therapeutic drug monitoring during hospitalization in 2022 (July 22—October 10).

Daily dosing of drugs, weeks 1–11	Week 1	Week 2	Week 3	Week 4	Week 5	Week 6	Week 7	Week 8	Week 9	Week 10	Week 11
*Olanzapine* Olanzapine Actavis	15 mg 40 ng/mL	20 mg	20 mg	20 mg	Tapered to 5 mg	Withdrawn	N.A.	N.A.	N.A.	N.A.	N.A.
*Carbamazepine* Tegretol CR	600 mg 31.8 μmol/L	600 mg	600 mg 31.1 μmol/L	600 mg	Withdrawn < 1.7 μmol/L	N.A. < 1.7 μmol/L	N.A.	N.A.	N.A.	N.A.	N.A.
*Lurasidone* Latuda	Titration to 74 mg	74 mg	148 mg	Reduced to 74 mg	74 mg < 10 ng/mL	74 mg 11 ng/mL	74 mg	74 mg 16.1 ng/mL	74 mg	74 mg	74 mg
*Clonazepam* Rivotril	N.A.	Titration to 1.5 mg	Reduced to 0.5 mg	Withdrawn	N.A.	N.A.	N.A.	N.A.	N.A.	N.A.	N.A.
*Clozapine* Leponex	N.A.	N.A.	N.A.	N.A.	Titration to 150 mg	200 mg 251 ng/mL	100 mg	75 mg	75 mg	100 mg	150 mg
*Valproate* Depakine Chrono	N.A.	N.A.	N.A.	600 mg	1000 mg 457 μmol/L	1000 mg	1000 mg 510 μmol/L	1000 mg 572 μmol/L	1000 mg	1000 mg	1000 mg

*Note:* *Carbamazepine* concentrations [μmol/L] were measured during weeks 1, 3, 5, and 6. *Lurasidone* plasma levels [ng/mL] were measured during weeks 5, 6, and 8. *Valproate* concentrations [μmol/L] were measured during weeks 5, 7, and 8. *Olanzapine* concentration was measured only during week 1 [ng/mL]. *Clozapine* plasma levels were measured only in week 6 [ng/mL]. Not applicable (N.A.) means that the drug was not given to the patient.

## 2 Discussion

Bipolar affective disorder with rapid cycling is challenging to treat because the evidence for pharmacological treatment is still sparse [1]. The Canadian Network for Mood and Anxiety Treatments (CANMAT) and the International Society for Bipolar Disorders (ISBD) recommend treating patients with bipolar disorder with rapid cycling by assessing thyroid function and discontinuing antidepressants, drugs of abuse, stimulants, and other psychotropic agents. Recommendations further state that lithium, valproate, olanzapine, and quetiapine all appear to have comparable maintenance efficacies in these patients [2].

Lurasidone was considered as an option for our patient since it is approved for the treatment of schizophrenia and bipolar depression [3] and because the patient's medical records documented that lithium, lamotrigine, olanzapine, quetiapine, and aripiprazole had little or no effect. Moreover, lurasidone is an antipsychotic agent with a low risk for sedation, weight gain, and adverse metabolic effects [3]. According to the British National Formulary (BNF 87, March–September 2024), the recommended starting dose is 37 mg q.d. or 18.5 mg q.d. when given with a moderate CYP3A4 inhibitor (e.g., verapamil). There is no guidance regarding the co‐administration of lurasidone with a CYP3A inducer, possibly because, according to the European SPC of Latuda, concomitant therapy of lurasidone with strong CYP3A4 inducers is contraindicated. US prescribing information for lurasidone also contraindicates such a combination. When drug interactions between lurasidone and various other drugs were studied in phase I and phase III trials, it was concluded that lurasidone should not be co‐prescribed with any strong CYP3A inducer or inhibitor and that dosage restrictions would be required when moderate CYP3A inhibitors were co‐administered. Importantly, dose adjustment for lurasidone was not required when co‐administered with valproate or lithium [4]. We did not find any specific description of drug interactions between carbamazepine and lurasidone in the literature.

The attending psychiatrist was not aware of this contraindication. The supervising psychiatrist recommended switching carbamazepine to valproate, olanzapine to clozapine, and monitoring lurasidone plasma levels. Some evidence suggests that clozapine may be both effective and relatively safe for resistant BP rapid cycling disorder [1]. In our case, withdrawal of carbamazepine increased lurasidone concentrations from < 10 ng q.d. to 16.1 ng/mL after 2–3 weeks, representing a 35% increase. Considering that the reference range of lurasidone concentrations is 15–40 ng/mL [5], the improvement of the patient's chronic bipolar disorder was likely attributable to the withdrawal of carbamazepine, which allowed lurasidone to reach therapeutic concentrations. However, the effect of valproate and clozapine cannot be excluded. Current recommendations indicate that lithium, valproate, olanzapine, and quetiapine have comparable maintenance efficacies [2]. This statement may be valid when making general comparisons of different drugs; however, in our specific case, it was not valid since lithium, olanzapine, and quetiapine were ineffective, while valproate was effective.

## Funding

This work was supported by COOPERATIO: Neurosciences program 207038, Charles University, Prague, Czech Republic.

## Data Availability

The data that support the findings of this study are available from the corresponding author upon reasonable request.

## References

[1] K. N. Fountoulakis , D. Kontis , X. Gonda , and L. N. Yatham , “A Systematic Review of the Evidence on the Treatment of Rapid Cycling Bipolar Disorder,” Bipolar Disorders 15, no. 2 (2013): 115–137. 23437958 10.1111/bdi.12045

[2] L. N. Yatham , T. Chakrabarty , D. J. Bond , et al., “Canadian Network for Mood and Anxiety Treatments (CANMAT) and International Society for Bipolar Disorders (ISBD) Recommendations for the Management of Patients With Bipolar Disorder With Mixed Presentations,” Bipolar Disorders 23, no. 8 (2021): 767–788. 34599629 10.1111/bdi.13135

[3] F. Corponi , C. Fabbri , I. Bitter , et al., “Novel Antipsychotics Specificity Profile: A Clinically Oriented Review of Lurasidone, Brexpiprazole, Cariprazine and Lumateperone,” European Neuropsychopharmacology: The Journal of the European College of Neuropsychopharmacology 29, no. 9 (2019): 971–985. 31255396 10.1016/j.euroneuro.2019.06.008

[4] Y. Y. Chiu , L. Ereshefsky , S. H. Preskorn , N. Poola , and A. Loebel , “Lurasidone Drug‐Drug Interaction Studies: A Comprehensive Review,” Drug Metabolism and Drug Interactions 29, no. 3 (2014): 191–202. 24825095 10.1515/dmdi-2014-0005

[5] G. Schoretsanitis , J. M. Kane , C. U. Correll , et al., “Blood Levels to Optimize Antipsychotic Treatment in Clinical Practice: A Joint Consensus Statement of the American Society of Clinical Psychopharmacology and the Therapeutic Drug Monitoring Task Force of the Arbeitsgemeinschaft für Neuropsychopharmakologie und Pharmakopsychiatrie,” Journal of Clinical Psychiatry 81, no. 3 (2020): 10‐4088. 10.4088/JCP.19cs13169 32433836

